# Comparison of Lipid Profiles Between Prediabetic and Non-prediabetic Young Adults

**DOI:** 10.7759/cureus.65251

**Published:** 2024-07-24

**Authors:** Irfan G Mulla, Ashish Anjankar, Ashok Shinde, Shilpa Pratinidhi, Sarita V Agrawal, Deepak B Gundpatil, Sandip D Lambe

**Affiliations:** 1 Biochemistry, Datta Meghe Institute of Higher Education and Research, Wardha, IND; 2 Biochemistry, Jawaharlal Nehru Medical College, Wardha, IND; 3 Physiology and Biochemistry, Sinhgad Dental College and Hospital, Pune, IND; 4 Biochemistry, Bharatratna Atalbihari Vajpayee Medical College, Pune, IND; 5 Biochemistry, Smt. Mathurabai Bhausaheb Thorat Institute of Medical Sciences and Research Center, Nashik, IND

**Keywords:** prediabetes risk, atherosclerosis, deranged lipid profile, cardiovascular complications, triglyceride, total cholesterol levels, ldl cholesterol

## Abstract

Introduction

Insulin resistance is considered a key component in the pathophysiology of prediabetes. Derangement in lipid parameters can occur in prediabetics that predispose to cardiovascular complications.

Material and methods

We performed an observational cross-sectional analytical study in a tertiary level Acharya Vinoba Bhave hospital, Sawangi, Wardha to compare the lipid profile in prediabetics with non-prediabetic young individuals between 18 and 35 age group in terms of parameters such as total cholesterol, triglyceride (TG), low-density lipoproteins (LDL) cholesterol, very low-density lipoprotein (VLDL) cholesterol, and high-density lipoprotein (HDL) cholesterol.

Results

We observed that prediabetics have significantly elevated total cholesterol, LDL cholesterol, VLDL cholesterol, and TG; and significantly reduced HDL cholesterol compared with the controls (p<0.001 each).

Conclusion

We conclude that the lipid parameters are deranged in prediabetics and this might contribute to the risk associated with dyslipidemia in this population.

## Introduction

Prediabetes is an intermediate stage of hyperglycemia in which all the adequate signs and symptoms have not yet developed to label a person as diabetic, but the glycemic parameters are borderline high [1]. The diagnosis of prediabetes is based on impaired fasting glucose (IFG) and the level of glycated hemoglobin (HbA1c). IFG is a condition in which the fasting blood glucose (FBG) lies between 100 mg/dL (5.6 mmol/L) and 125 mg/dL (6.9 mmol/L). Similarly, the level of HbA1c lies between 5.7% and 6.4% (39-46 mmol/mol) for prediabetic subjects [2]. Prediabetes has been shown to predispose to diabetes mellitus [1].

Prediabetes has been conventionally considered benign; however, it is shown to have a strong association with microvascular and macrovascular complications [3]. Observational evidence has shown the association of prediabetes with various diabetic complications such as nephropathy, neuropathy, retinopathy as well as cardiovascular complications, which is further exacerbated by an elevated potential for atherosclerosis. Although a part of the risk for macrovascular complications can be attributed to the natural progression of the disease to diabetes, nevertheless, studies have suggested independent concern for complications even in those who do not eventually progress to diabetes [4].

Prediabetes has been associated with insulin resistance and β-cell dysfunction, both of which can be present even before the changes in blood glucose are detectable [5]. Insulin resistance is considered a key component in the pathophysiology of prediabetes. Increases in serum triglyceride (TG) levels have been linked with reduced insulin secretion and β-cell dysfunction in prediabetics [6]. Literature has reported that raised visceral adipose tissue (VAT) levels are linked with higher lipolytic activity that releases adipokines and fatty acids into the portal circulation, resulting in insulin resistance in the liver [7-9]. From a mechanistic point of view, the induction of insulin secretion by glucose via the fatty acid cycle can be reduced by hypertriglyceridemia, and the apoptotic death of β-cells can be promoted by enhancing the production of nitric oxide and ceramide. Also, increases in TG levels can enhance lipotoxicity by their accumulation within the beta cells of the islets [10].

Insulin resistance has been reported to be associated with an atherogenic dyslipidemic profile and a proinflammatory state. Dyslipidemia in insulin-resistant individuals is characterized by elevated TGs, apolipoprotein B, small dense low-density lipoprotein (LDL) particles, and reduced high-density lipoprotein (HDL) concentration and smaller HDL particle size.

It has been suggested that elevated cholesterol levels predispose to atherosclerosis. Cholesterol is a key component of the atheromatous plaques that clog the blood vessels and lead to vascular occlusion and ischemia of the involved organs, including myocardial infarction [11]. Therefore, determining derangements in the lipid profile at the stage of prediabetes itself might provide a clue regarding the future development of cardiovascular complications, and thus, might provide an opportunity to introduce interventions at this stage.

We, therefore, envisaged a study to enumerate the lipid profile in prediabetics in comparison with normal healthy individuals, to determine whether the lipid parameters in the prediabetics are deranged.

## Materials and methods

We performed an observational cross-sectional analytical study in a tertiary-level Acharya Vinoba Bhave hospital in Sawangi Meghe, India.

Study population

The study was conducted on 142 prediabetic young individuals, screened from the tertiary level Acharya Vinoba Bhave hospital and a similar group of 142 non-prediabetic young individuals identified from the same population. The following patients were excluded: type 1 diabetes mellitus, type 2 diabetes mellitus, hepatic diseases apart from non-alcoholic steatosis, kidney disease, and pregnant women. The study was conducted from January 2023 to April 2024.

Prediabetic young individuals were taken as cases while non-prediabetic young individuals were the control group in this study. After obtaining the requisite permission from the Ethics Committee of the Institute, all patients attending our tertiary care center were approached for participation. They explained the methodology and consent in writing was obtained from those ready to participate. Their body weights, heights, body mass index (BMI), and waist circumference were recorded.

Inclusion criteria

1) Age from 18 to 35 years, 2) FBG levels between 100 125 mg/dL and 125 mg/dL, and 3) HbA1c between 5.7% and 6.4%.

Exclusion criteria 

1) Type 1 diabetes mellitus, 2) type 2 diabetes mellitus, 3) hepatic disease, 4) patients on insulin, thiazolidinediones, or metformin, 5) any malignancies, 6) other diseases and drugs including corticosteroids, octreotide, beta blockers, thiazide diuretics, statins, and antipsychotics that alter glucose metabolism, and 7) pregnant women.

The screening questionnaire was also approved by the Ethics Committee of the Institute. The recruited participants were classified into prediabetics and healthy controls based on the HbA1c and FBS as per American Diabetes Association (ADA) guidelines. Prediabetics were defined as those with HbA1c values between 5.7% and 6.4%, FBS between 100 mg/dL and 125 mg/dL, and not on any antidiabetic treatment.

Study parameters

Blood samples were collected by a vein puncture after 10-12 h of fasting, and the blood sample (5 mL) was split into two aliquots for further testing and analysis. The first aliquot (2 mL) was dispensed into a tube containing ethylene-diamine tetra-acetic acid (EDTA), removed from the tube EDTA, and then processed in less than three hours and utilized for HbA1c measurement. The second aliquot (remaining blood (3 mL)) was transferred to a plain tube, and after clotting, the tube was centrifuged (3000 rpm for 15 min) to gather serum and assess for total cholesterol, TGs, and HDL using Cobas c311 analyzer (Cobas-Roche, Germany) preloaded with the respective reagent kits. Serum very low-density lipoprotein (VLDL)-cholesterol was computed using Friedewald’s equation: VLDL-cholesterol=TG/5. Similarly, serum LDL-cholesterol is computed using the equation: LDL-cholesterol=total cholesterol-(HDL-cholesterol+VLDL-cholesterol).

Sample size calculation

The sample size calculated per arm of our study was 142, considering an alpha error of 0.05, power of 80%, and r=0.5, derived from the study by Hussein and co-workers [12]. Hence, the total sample size for the two study arms was 284.

Statistical analyses

The demographic data were descriptively reported as mean±SD. The parameters with quantitative data were assessed with the Student t-test after confirmation of its normality. All the statistical tests were accomplished with the SPSS software's latest version 24.0 (IBM Corp., Armonk, NY).

## Results

Age

The age of cases in our study was found to be 27.74±4.98 years, while that of controls was found to be 27.77±5.00 years (Table 1). The ages of the two groups were found to be similar when compared with the t-test (p=0.96) (Table 1).

**Table 1:** Ages in the two study arms p<0.05 is considered statistically significant.

Group	Mean±SD (years)	T score	P-value
Cases	27.74±4.98	-0.05	0.96
Controls	27.77±5.00		

Body weight

The mean body weight of cases was found to be 84.84±5.61 kg, while that of controls was found to be 66.62±5.51 kg (Table 2). The cases were found to have significantly greater body weights than the controls (p<0.001) (Table 2).

**Table 2:** Body weights in the two study arms ^*^statistical significance and p<0.05 is considered statistically significant.

Group	Mean±SD (kg)	T score	P-value
Cases	84.84±5.61	27.61	<0.001*
Controls	66.62±5.51		

Height

The mean height of cases was found to be 163.75±4.65 cm, while that of controls was found to be 163.72±4.72 cm (Table 3). The heights of the two groups were observed to be similar (p=0.07) (Table 3).

**Table 3:** Heights in the two study arms p<0.05 is considered statistically significant.

Group	Mean±SD (cm)	T score	P-value
Cases	163.75±4.65	1.85	0.07
Controls	163.72±4.72		

Waist circumference

The mean reading of waist circumference of cases was found to be 41.82±2.53 cm, while that of controls was found to be 34.38±1.88 cm (Table 4). The cases were found to have significantly greater waist circumferences than the controls (p<0.001) (Table 4).

**Table 4:** Waist circumferences in the two study arms ^*^statistical significance and p<0.05 is considered statistically significant.

Group	Mean±SD (cm)	T score	P-value
Cases	41.82±2.53	28.13	<0.001*
Controls	34.38±1.88		

Body mass index

The average BMI of cases was found to be 31.69±2.50 kg/m^2^, while that of controls was found to be 24.84±1.56 kg/m^2^ (Table 5). The cases were found to have significantly greater BMIs than the controls (p<0.001) (Table 5).

**Table 5:** BMIs in the two study arms ^*^statistical significance and p<0.05 is considered statistically significant. BMI: body mass index

Group	Mean±SD (kg/m^2^)	T score	P-value
Cases	31.69±2.50	27.70	<0.001*
Controls	24.84±1.56		

Total cholesterol

**Figure 1:**
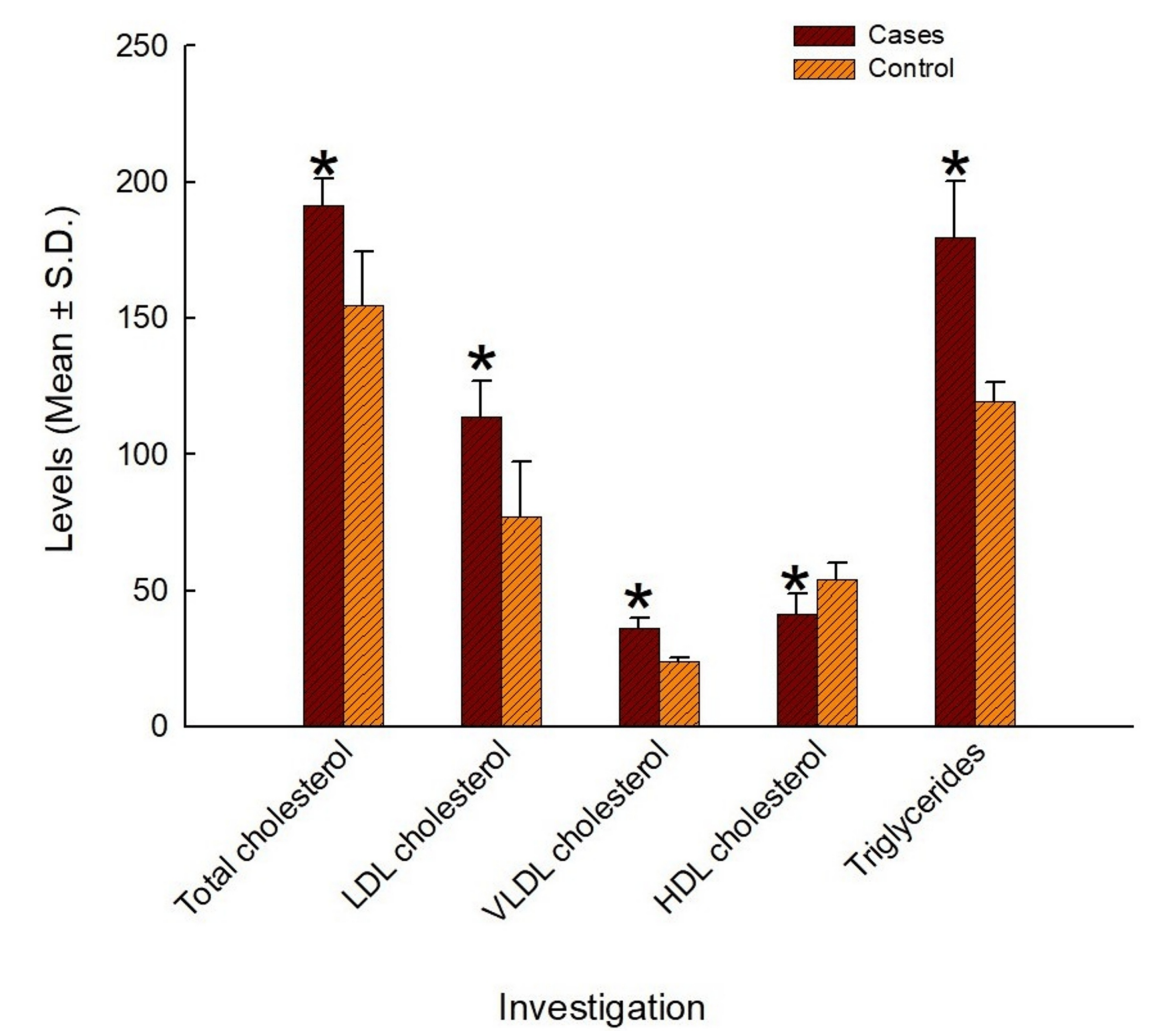
Lipid parameters in the two study arms ^*^statistical significance and p<0.05 is considered statistically significant. LDL: low-density lipoproteins; VLDL: very low-density lipoprotein; HDL: high-density lipoprotein; TG: triglyceride

The total cholesterol of the cases was found to be 191.25±9.98 mg/dL, while that of controls was found to be 154.64±19.84 mg/dL (Table 6, Figure 1). The total cholesterol levels of the two groups were observed to be significant on the t-test (p<0.001), with the cases showing significantly greater total cholesterol levels than the controls (Table 6).

**Table 6:** Lipid parameters in the two study arms ^*^statistical significance and p<0.05 is considered statistically significant. LDL: low-density lipoproteins; VLDL: very low-density lipoprotein; HDL: high-density lipoprotein; TG: triglyceride

Parameter	Cases (mean±SD)	Controls (mean±SD)	T score	P-value
Total cholesterol (mg/dL)	191.25±9.98	154.64±19.84	19.683	<0.001*
LDL cholesterol (mg/dL)	113.99±12.85	76.89±20.40	18.337	<0.001*
VLDL cholesterol (mg/dL)	35.93±4.11	23.91±1.36	33.086	<0.001*
HDL cholesterol (mg/dL)	41.33±7.41	53.84±6.23	-15.399	<0.001*
TG (mg/dL)	179.63±20.53	119.57±6.82	33.083	<0.001*

Low-density lipoprotein cholesterol

The LDL cholesterol of the cases was found to be 113.99±12.85 mg/dL, while that of controls was found to be 76.89±20.40 mg/dL (Table 6, Figure 1). The LDL cholesterol levels of the two groups were observed to be significantly different on the t-test (p<0.001), with the cases showing significantly greater LDL cholesterol levels than the controls (Table 6).

Very low-density lipoprotein cholesterol

The mean VLDL cholesterol of the cases was found to be 35.93±4.11 mg/dL, while that of controls was found to be 23.91±1.36 mg/dL (Table 6, Figure 1). The VLDL cholesterol levels of the two groups were observed to be significantly different on the t-test (p<0.001), with the cases showing significantly greater VLDL cholesterol levels than the controls (Table 6).

High-density lipoprotein cholesterol

The HDL cholesterol of the cases was found to be 41.33±7.41 mg/dL, while that of controls was found to be 53.84±6.23 mg/dL (Table 6, Figure 1). The HDL cholesterol levels of the two groups were observed to be significantly different on the t-test (p<0.001), with the cases showing significantly lesser HDL cholesterol levels than the controls (Table 6).

Triglycerides

The TG levels of the cases were found to be 179.63±20.53 mg/dL, while that of controls was found to be 119.57±6.82 mg/dL (Table 6, Figure 1). The TG levels of the two groups were observed to be significantly different on the t-test (p<0.001), with the cases showing significantly greater TG levels than the controls (Table 6).

## Discussion

We observed that the prediabetics in our study demonstrated significantly elevated total cholesterol, LDL cholesterol, VLDL cholesterol, and TG, and significantly reduced HDL cholesterol compared with the controls.

Our observations are similar to those reported in the literature. In a study conducted in India on 124 prediabetics and 101 healthy controls by Kansal and Kamble, the total cholesterol, LDL cholesterol, VLDL cholesterol, TG, and LDL/HDL ratio were significantly elevated in the prediabetics, while the HDL cholesterol was significantly lower among the prediabetics, similar to what we have observed in our study [13].

Similarly, a retrospective study conducted on 239 medical records in India by Chakraborty et al. has also reported that total cholesterol, TG, and LDL cholesterol, as well as other atherogenic indices such as atherogenic coefficient, Castellis risk index, and atherogenic index were significantly increased in prediabetics, while HDL cholesterol was significantly decreased in prediabetics [14]. These findings also corroborate the observations in our study.

Moreover, Jasim et al. have verified the role of lipid parameters in distinguishing between prediabetics and normal healthy individuals by constructing a receiver operating characteristics (ROC) curve, which showed reasonably good sensitivity, specificity, and AUC values for the same [15]. In fact, some studies have also correlated the elevations in TGs with an increased risk of cardiovascular disorders [16-18].

Additionally, Tirosh et al. have reported a 4% increase in the risk for progression to diabetes mellitus with every 10 mg/dL increase in the TG levels [19]. Thus, a deranged lipid profile in prediabetics may additionally increase the risk of progression to type 2 diabetes mellitus as well.

A deranged lipid profile indicates a greater risk of developing atherosclerosis and subsequent cardiovascular outcomes. Elevated levels of cholesterol are considered to be one of the factors that explain the predisposition of prediabetics to cardiovascular complications. Hence, there might be a case for addressing the lipid values, so as to reduce the risk for atherosclerosis. In a study by Barua, treatment of the deranged lipid profile with medications such as metformin and a dipeptidyl peptidase 4 (DPP-IV) inhibitor in combination with lifestyle changes led to significant reductions in HbA1c levels, serum TGs, and BMI values [20].

Limitations 

Our study also has a few limitations. Although we enrolled a good number of participants (284), ours was a single-center study. A multi-center study would have yielded better information as to whether the difference observed in our study is consistent across multiple geographical regions and study populations. Moreover, we have restricted our evaluation to a lipid profile. Determination of other confounders such as smoking, alcoholism, diet, and other co-morbidities could have provided a more robust correlation between the lipid profile and complications among the prediabetics.

## Conclusions

Based on our observations, we conclude that the lipid parameters in prediabetics are deranged compared with the normal healthy population toward the risk of dyslipidemia. This provides us a possible opportunity that treatment of the lipid profile and control of the lipid parameters might reduce the risk of dyslipidemia in these persons. Further studies are needed on whether improvements in lipid profiles in prediabetics actually translate to a reduction in cardiovascular complications.
